# Using the Developmental Gene *Bicoid* to Identify Species of Forensically Important Blowflies (Diptera: Calliphoridae)

**DOI:** 10.1155/2013/538051

**Published:** 2013-03-18

**Authors:** Seong Hwan Park, Chung Hyun Park, Yong Zhang, Huguo Piao, Ukhee Chung, Seong Yoon Kim, Kwang Soo Ko, Cheong-Ho Yi, Tae-Ho Jo, Juck-Joon Hwang

**Affiliations:** ^1^Department of Legal Medicine, College of Medicine, Korea University, 126-1 Anamdong 5-ga, Seongbuk-gu, Seoul 136-705, Republic of Korea; ^2^Department of Anesthesiology and Pain Medicine, CHA University Medical College, 43 Beolmal 30th-road, Gyeonggi-do, Seongnam 463-836, Republic of Korea; ^3^Beijing Scales Forensic Center, 100 Chaoyangru, Chaoyang-qu, Beijing 100025, China; ^4^Institute of Environmental Ecology, College of Science and Technology, Korea University, 126-1 Anamdong 5-ga, Seongbuk-gu, Seoul 136-705, Republic of Korea; ^5^Department of Science Education, Chinju National University of Education, 380 Shinandong, Gyeongsangnam-do, Jinju 660-756, Republic of Korea

## Abstract

Identifying species of insects used to estimate postmortem interval (PMI) is a major subject in forensic entomology. Because forensic insect specimens are morphologically uniform and are obtained at various developmental stages, DNA markers are greatly needed. To develop new autosomal DNA markers to identify species, partial genomic sequences of the *bicoid* (*bcd*) genes, containing the homeobox and its flanking sequences, from 12 blowfly species (*Aldrichina grahami, Calliphora vicina, Calliphora lata, Triceratopyga calliphoroides, Chrysomya megacephala, Chrysomya pinguis, Phormia regina, Lucilia ampullacea, Lucilia caesar, Lucilia illustris, Hemipyrellia ligurriens* and *Lucilia sericata*; Calliphoridae: Diptera) were determined and analyzed. This study first sequenced the ten blowfly species other than *C. vicina* and *L. sericata*. Based on the *bcd* sequences of these 12 blowfly species, a phylogenetic tree was constructed that discriminates the subfamilies of Calliphoridae (Luciliinae, Chrysomyinae, and Calliphorinae) and most blowfly species. Even partial genomic sequences of about 500 bp can distinguish most blowfly species. The short intron 2 and coding sequences downstream of the *bcd* homeobox in exon 3 could be utilized to develop DNA markers for forensic applications. These gene sequences are important in the evolution of insect developmental biology and are potentially useful for identifying insect species in forensic science.

## 1. Introduction

DNA-based methods in forensic entomology have advanced rapidly since the first successful attempt to discriminate important forensic insect species in 1994 [1]. DNA genotyping is now routinely used in the laboratory [2]. Traditionally, a small number of expert insect taxonomists have identified species based on morphological traits. However, growth rates and habitats of important forensic insects differ from one species to another. Further, insect specimens captured at the crime scene or from corpses are often at immature developmental stages and are outwardly indistinguishable, making them morphologically unsuitable for forensic purposes [3]. Identifying important forensic insects, including blowflies and fleshflies, using DNA-based techniques is promising because it contributes considerably to the estimation accuracy of the postmortem interval (PMI) [4]. 

 Blowfly species (Diptera: Calliphoridae) are among the first to colonize decomposing human bodies, and they play a significant ecological role by infesting a corpse with their larvae. Many blowfly species are distributed globally, while others show more localized distributions. Blowflies have 12 chromosomes, including ten autosomes and X and Y chromosomes [5]. 

DNA-based techniques for identifying forensic blowfly species have been developed using genes encoded in mitochondrial genomes, such as cytochrome oxidase subunits I and II (COI and COII) [5–11] or nuclear genes such as 28S ribosomal RNA [12, 13]. Since the conventional DNA markers have some limitations, new autosomal-based DNA markers for the identification of forensic insect species may ameliorate the problems associated with conventional markers. Among blowfly species, two Lucilia species (*L. illustris* and caesar), two tropical Luciliinae species (*Lucilia cuprina* and *Hemipyrellia ligurriens*), and two African *Chrysomya* species (*C. putoria* and *C. chloropyga*) show species level paraphylies [14–16]. According to Zaidi et al., identification of closely related species may demand a multigene approach [17].

*Bicoid* (*bcd*) is a gene whose transcripts are secreted from maternal nurse cells into the anterior poles of eggs. During embryonic patterning, the protein forms a concentration gradient that determines the anterior-posterior axis, significantly impacting the development of the head and thorax in *Drosophila melanogaster* [18, 19]. The bcd protein is a transcription and translation factor that contains a homeodomain and that acts as a morphogen in the formation of the anterior-posterior pattern [20]. Embryonic *bcd* transcripts localize in the anterior regions of blowfly and housefly embryos [21, 22].

 Found only in higher dipteran families (suborder: Cyclorrhapha), which include most forensic fly species, *bcd* genes are evolutionary novel [20, 23]. The gene *bcd* is an exceptional member of the *Hox3 *cluster of homeobox genes [23]. Full or partial *bcd* gene sequences have been determined in many dipteran species, including a primitive cyclorrhaphan fly (*Megaselia abdita*) [23], a dozen *Drosophila* species [24], the housefly (*Musca domestica*), and some blowflies, such as *Lucilia sericata* and *Calliphora vicina* [25]. A genomic map of the housefly *bcd* gene is now available, along with that of *D. melanogaster* [25]. Though *bcd* expression in housefly and blowflies may have diverged from that of the fruitfly [21, 22, 26], the structures of the four exons have been largely conserved between the fruitfly and housefly, with some introns expanded in housefly [25] (see supplementary Figure 1 in Supplementary Material available online at http://dx.doi.org/10.1155/2013/538051). 

 To evaluate the applicability of *bcd* gene sequencing to the identification of forensic insect species, partial genomic sequences of *bcd* genes containing the homeobox, and flanking sequences were obtained by PCR amplification from a number of specimens of 12 blowfly species collected in Korea. The sequences were determined and analyzed for suitability in blowfly species identification.

## 2. Materials and Methods

### 2.1. Fly Collection

To collect flies, pork liver bait was used to attract them to traps in several regions of South Korea. After being submerged in 70% ethanol solution, flies were identified under a dissecting microscope. Morphological identification was done under the taxonomic Keys by Kano and Shinonaga [27]. Both male and female flies were subjected to DNA analysis. To trace the transmission of single nucleotide and insertion/deletion polymorphisms, some adult blowfly species were mated in the laboratory and genotyped. A few F1 and several F2 progenies of the same first-generation females were obtained and subjected to subsequent experimental analysis. 

### 2.2. DNA Extraction

After immersion into liquid nitrogen, whole blowfly bodies without heads were ground into powder in 1.5 mL microcentrifuge tubes with piston pellets (Tokken Inc., Japan). The genomic DNA was extracted with phenol-chloroform-isoamyl alcohol (25 : 24 : 1). 

### 2.3. Polymerase Chain Reaction (PCR) and Cloning

The initial PCR was performed with a degenerate primer pair (F1 and R1) based on similarities in the *C. vicina* (AJ297855), *L. sericata* (AJ297856), and *M. domestica* (AJ297853 and AJ297854) coding sequences (see supplementary Table 1). The initial degenerate primer pair amplified–350 bp-long homologous sequences, spanning from the 3′ region of exon 2 to immediately downstream of the *bcd* homeobox motif. For the initial PCR, touch-down amplification was performed with an initial step of 95°C for 11 min, followed by eight cycles of 95°C for 30 sec, annealing temperatures starting at 42°C for 1 min and decreasing 1°C/cycle, and extension at 72°C for 1 min. These cycles were followed by 35 cycles of 95°C for 30 sec, 42°C for 1 min, and 72°C for 1 min, with a final extension at 72°C for 15 min. DNA fragments amplified with degenerate primers were visualized on agarose gel electrophoresis, eluted using a GeneClean III kit (Q-Biogene, Bio 101 Systems, Carlsbad, CA, USA), and cloned into a TA vector system (Real Biotech Corporation, Banquiao, Taiwan). The plasmids were extracted from the *E. coli* host strains, and the sequences were determined. 

Based on the initial sequences, two new 5′ primers (F2 and F3) were designed to hybridize 60–80 bp downstream of the initial 5′ primer (F1). The new 5′ primers and a 3′ primer (R2), which hybridized more than 200 bp downstream of the initial primer, were used for subsequent PCR amplifications [9, 10]. After analyzing the sequences from the first and second rounds of PCR, specific primers (FD1–9 and RD1–3) were designed (see supplementary Table 2) and used to amplify 500–525 bp targets. The sequences were determined either by direct sequencing or by sequencing after cloning. 

### 2.4. Sequencing

Sequencing was performed with automatic sequencers (ABI PRISM 310 genetic analyzer) [9, 10]. Direct sequencing of amplified products was performed using the BigDye Terminator Sequencing kit (v1.1) (Applied Biosystems, Foster City, CA, USA). The specific forward and reverse primer sets (FD1–9 and RD1–3) were used to sequence the amplified products. Some amplified DNAs were cloned into TA vectors, and multiple colonies were sequenced to delineate haplotypes. All of the sequences obtained with the FD-RD primer pairs were submitted to GenBank (Accession Numbers GU256065–256168 and 979851–979856). Most *bcd* sequences were heterozygous though homozygous sequences of two *L. sericata* individuals (GU256104-5 and GU256107-8), one *T. calliphoroides* (GU256113-4), and two *Ch. pinguis* (GU256138–141) were also deposited. 

### 2.5. Sequence Analysis and Phylogenetics

 mRNA and genomic sequences were downloaded from GenBank (NCBI) and analyzed in order to resolve the *bcd* genomic structures (see supplementary Figure 1). The XM_00210237 and AF465792 sequences were downloaded to resolve the genomic structure of *Drosophila simulans bcd,* which has about 97% nucleotide identity to the coding sequence of* D. melanogaster*. For the common housefly (*M. domestica*), the AJ297853 and AJ297854 mRNA sequences were compared to partial genomic sequences (AJ297850–52). The *bcd *sequences of the housefly and the mRNA sequence of *C. vicina* (AJ297855) were used as references to compare and analyze new blowfly DNA sequences. 

 Nucleotide and amino acid sequences were aligned using ClustalW in MEGA4 software [28, 29], which calculated nucleotide distance matrices based on maximum composite likelihoods. Phylogenetic trees were generated using the neighbor-joining method with a bootstrapping of 1,000 replicates. 

## 3. Results

### 3.1. Coding Sequence Variation

 The sequences were analyzed according to a dichotomy of coding (3′ region of exon 2 and 5′ half of exon 3) and noncoding sequences (intron 2) (Table 1). The coding sequences, excluding intron 2, showed a relatively small intraspecific distance. *Ch. pinguis *and *L. ampullacea* individuals showed similar variation (0.0083, *n* = 7 and 0.0081, *n* = 3; resp.), while *A. grahami*, *Ch. megacephala*, *L. illustris,* and *H. ligurriens *had virtually no intraspecific variation (Table 1).

 The interspecific nucleotide differences (distances) in the coding sequences between the 12 blowflies ranged from 0.21  to  0.26 (see supplementary Table 3). Among the 12 blowfly species, *C. vicina* and *Ch. pinguis* differed the most (distance = 0.1235) followed by *C. vicina* and *Ch. megacephala *(0.1215). On the other hand, *L. caesar *and *L. illustris *(distance = 0.0006) showed the highest sequence similarity, followed by *Ch. megacephala *and *Ch. pinguis *(0.01646). 

**Figure 1:**
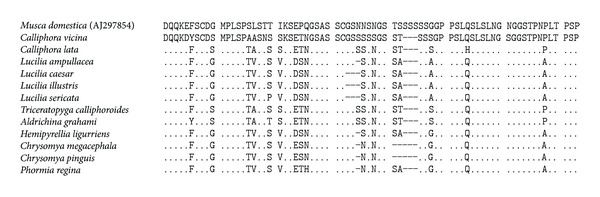
Multiple alignments of the amino acid sequences downstream of the *bcd* homeodomain in blowflies. The sequences–70 amino acids downstream of the *bcd* homeodomain in 12 blowfly species were aligned to the 73 amino acid sequence of *M. domestica* (AJ297854). All 12 blowfly species lack the three serine residues present in *M. domestica*. Other serine residues are also deleted from around the distinct serine-rich region. All blowfly sequences were represented without being accountant for intraspecific variation.

 Partial amino acid sequences downstream of the *bcd* homeodomain (about 70 amino acids) displayed comparatively higher inter- and intraspecific variations than did the homeodomains among the 12 blowfly species (Figure 1). These variations reflect some nonsynonymous nucleotide and codon insertion/deletion polymorphisms. The partial protein sequence of exon 2 (about 25 amino acids) showed few amino acid changes among and within species. 

### 3.2. Intron 2 Variation

Intron 2 from *bcd* genes showed high interspecific variation in the lengths and nucleotide sequences. The sequence lengths could be classified into two types (Table 2). The first type was similar in size to *D. melanogaster *(55 bp). *bcd* intron 2 was either 52 bp or 53 bp in *T. calliphoroides*, 53 bp in *A. grahami*, *C. vicina*, *C. lata*, *L. caesar*, and *L. illustris*, 53–55 bp in *L. sericata* and 57 bp in *H. ligurriens*. Intron 2 in *L. ampullacea* was either 53 bp or 60 bp depending on the presence of an eight-nucleotide insertion. These blowfly species belong to the subfamilies Calliphorinae and Luciliinae. In addition, a multiple alignment showed that intron 2 from Calliphorinae species, such as *T. calliphoroides*, *A. grahami*, *C. vicina*, and *C. lata*, differed from those of the Luciliinae species, such as *H. ligurriens*, *L. caesar*, *L. illustris*, *L. ampullaceal*, and *L. sericata *(Table 2).

The second type of intron was about 10 bp longer, similar in size to that of the housefly (*M. domestica*) (73 bp). Intron 2 was 63 bp in *P. regina*, 68-69 bp in *Ch. pinguis*, and 69 bp in *Ch. megacephala*. These species belong to the subfamily Chrysomyinae. 

 The interspecific sequence variation in intron 2 was relatively higher than that of the coding sequences (see supplementary Table 4). The interspecific distance was highest between *A. grahami* and *P. regina* (0.9730). *L. caesar* and *L. illustris* (distance = 0.0263) showed the highest similarity, with the next highest observed between *Ch. megacephala* and *Ch. pinguis* (distance = 0.0319) (see supplementary Table 4).

Six species,* Ch. pinguis*, *L. ampullacea*, *L. sericata*, *L. illustris*, *L. caesar* and *C. lata*, contained intraspecific single nucleotide polymorphisms (Table 2). Single-nucleotide insertion/deletion (indel) polymorphisms were discovered in *Ch. pinguis*, and *T. calliphoroides*, while a two-nucleotide indel polymorphism was found in *L. sericata*. An eight-nucleotide indel polymorphism was also discovered in *L. ampullacea*. *L. sericata,* grown in the laboratory, confirmed the stable transmission of these single-nucleotide and indel polymorphisms to progeny (data not shown). 

### 3.3. Phylogenetic Tree

**Figure 2:**
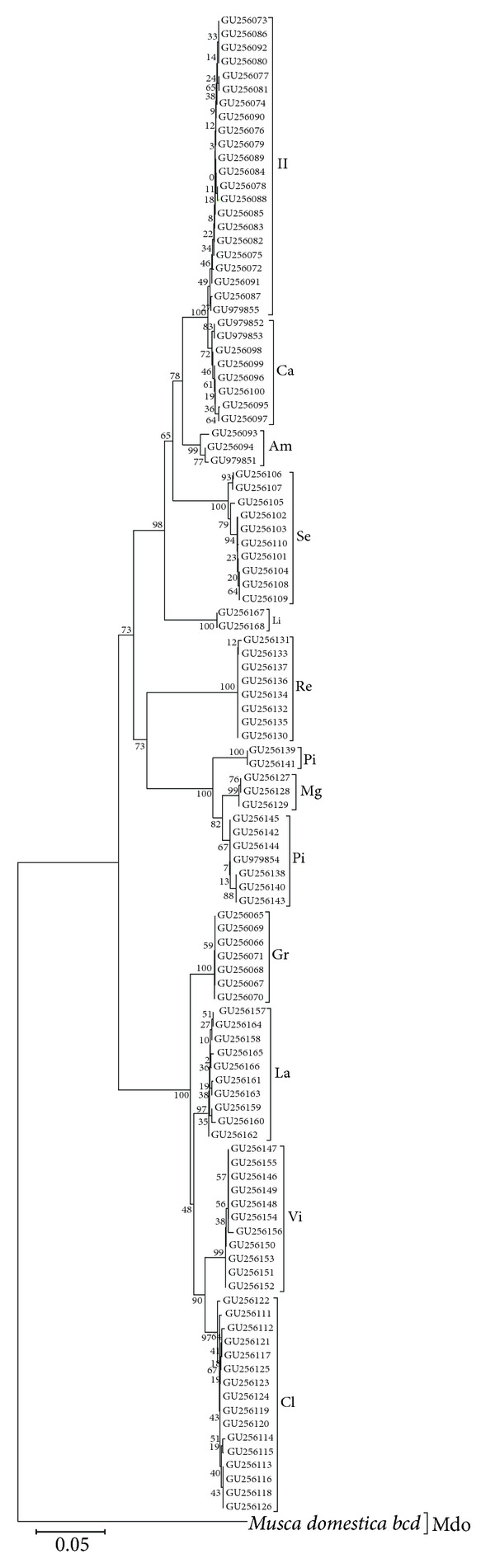
A neighbor-joining phylogenetic tree of 12 blowfly species. The *bcd *genomic sequence of *M. domestica *was used as the outgroup to construct the phylogenetic tree. Most sequences are diploid except some haploid sequences of *L. sericata* (GU256104-5, GU256107-8), *T. calliphoroides *(GU256113–GU256114) and *Ch. pinguis* (GU256138-141).

 A phylogenetic tree of the 12 blowfly species was constructed based on the partial *bcd *genomic sequences containing the homeobox and flanking coding and noncoding sequences (Figure 2). With the partial *bcd* genomic sequences, three subfamilies: Luciliinae, Chrysominae, and Calliphorinae, within the family Calliphoridae, could be classified among the 12 blowfly species collected from Korea. In the subfamily Luciliinae, the taxonomic status of the genus *Hemipyrellia* (*H. ligurriens*), which is distinct from the genus *Lucilia* (*L. illustris*, *L. caesar*, *L. ampullacea*, and *L. sericata*), was confirmed. In the subfamily Chrysominae, some *Ch. pinguis* haplotypes fell outside a cluster of other *Ch. pinguis* and *Ch. megacephala* sequences. In the subfamily Calliphorinae, the genus *Calliphora*, namely, *C. vicina* and *C. lata* in this study, did not form a monophyletic group, clustering with a species of a different genus, *A. grahami*. Almost all blowfly species in this study could be discriminated in the phylogenetic tree except the *Ch. megacephala* and *Ch. pinguis* species pair (Figure 2).

## 4. Discussion

The genomic *bcd* sequence is potentially useful for identifying forensically important insect species. Partial *bcd* sequences of about 500 bp were amplified to evaluate possible species-specific DNA markers, with promising results. 

We observed intra- and interspecific lengths and sequence polymorphisms in intron 2 *bcd* from 12 blowfly species. The sizes of intron 2 in *D. melanogaster*, *D. simulans*, and *M. domestica *were interesting (Figure 1). Among the three dipteran species, intron 2 showed small-size differences (55 bp, 55 bp, and 73 bp, resp.). In contrast, introns 1 and 3 showed substantial size differences and were expanded in *M. domestica*, but conserved to a considerable extent in the two *Drosophila* species. Introns 1 and 3 were expanded 6–8 times in *M. domestica* (12–13 kbp and 2.5 kbp) compared to those of the two *Drosophila* species (about 400–500 bp) (Figure 1) [25]. 

 The *D. melanogaster bcd* sequences (AF466621-45) did not vary in intron 2 of *bcd* (Table 2) among 25 isofemale lines collected from Zimbabwe, a representative ancestral population [30]. Intron 2 in *M. domestica* is larger than that of *D. melanogaster* and showed intraspecific variation that transmits stably to ensuing generations (data not included). 

The interspecific length polymorphisms of intron 2 could be classified into two categories: first, a D type including the subfamilies Calliphorinae and Luciliinae, similar to the intron 2 of *D. melanogaster* and *D. simulans*, and second, an M type including the subfamily Chrysomyinae, similar to that of *M. domestica* (Table 2). Lengthwise, the first type could be further classified into the two subfamilies: intron 2 in Luciliinae tended to be slightly longer than Calliphorinae (Table 2). The nucleotide distance values complement the length polymorphism-based distinction: species within the same subfamily showed below-average pairwise distances, while species of different subfamilies displayed above-average pairwise distances (average distances among the 12 blowfly species = 0.5453) (see supplementary Table 4).

 Downstream of the *bcd* homeobox (about 210 nucleotides), exon 3 also displayed intra- and interspecific variations (Figure 1). The posthomeodomain sequence, which includes exons 3 and 4, is thought to interact with other transcription factors and proteins for translation [20]. 

There are also interspecific amino acid differences immediately downstream of the homeobox (Figure 1), which may provide the basis for a simple, reliable, and effective DNA-based method for distinguishing forensic insect species. Thus, taxon-specific sequence-characterized amplified region (SCAR) markers [31–33] based on *bcd* will likely become available for forensic applications. 

The SCAR markers can be developed to discriminate between a pair of sister species like *L. caesar *and *L. illustris* [15, 27]. In this study, eight *L. caesar* individuals and 22 *L. illustris* individuals were subjected to analysis of *bcd* polymorphisms. A single nucleotide difference between *L. caesar *and *L. illustris* (position 42 in intron 2, multiple alignments in Table 2) could distinguish the two closely related species; cytosine (C) is present in this location in *L. caesar* and adenine (A) in *L. illustris*. This specific difference can be incorporated into SCAR markers for species identification. These markers should be able to discriminate blowfly species captured in South Korea. 

All blowfly species were well clustered according to subfamilies Luciliinae, Chrysominae and Calliphorinae in this study (Figure 2), according to partial *bcd* sequencing phylogenetic analyses. In the subfamily Luciliinae, the genera *Hemipyrellia* and *Lucilia* formed individual clades. The closely related sister species, *L. illustris* and *L. caesar*, were also distinguishable. In the subfamily Chrysominae, the analysis of more individuals may be necessary to distinguish between *Ch. megacephala* and *Ch. pinguis*. A greater number or complete *bcd* sequences from more individuals may resolve the apparent species-level paraphyly in *Ch. pinguis*. The genus-level paraphyly in the subfamily Calliphorinae (genera *Aldrichina*, *Cynomya*, *Eucalliphora, Triceratopyga*, etc.) based on mtDNA phylogenies has been reported previously [10, 34, 35]. *C. vicina* and *C. lata* showed similar trends in this study; they did not form a genus-level monophyly, instead *A. grahami* clustered with the two *Calliphora* species (Figure 2). Based on the COI phylogeny, Rognes [34] and Whitworth [35] suggested that *A. grahami *and the genus *Eucalliphora* should be incorporated into the genus *Calliphora*. The *bcd* phylogenetic tree supports this suggestion, calling for a possible future taxonomic revision.

This study showed that even a 500 bp partial bcd genomic sequence containing the homeobox motif can be useful for distinguishing most forensically important blowfly species including previously recognized confused taxa, that is, *L. illustris* and *L. caesar*. Additionally, this kind of study may be extended to other confused taxa such as *L. cuprina* and some *Chrysomya* species [14–16]. This study is the first to use a renowned developmental gene, *bicoid*, in forensic science and entomology. Bcd sequences of 12 blowfly species are useful for species identification although many blowfly species uncommon in Korea were not included in this study. However, we expect that other researchers can easily apply our degenerate PCR strategy to amplify *bcd* sequences of their regional blowfly species.

## Supplementary Material

Supplementary Table 1: The primers used for the initial and second PCR amplification.
Supplementary Table 2: The species-specific primer pairs used.
Supplementary Table 3: Inter-specific distances of the *bcd* coding sequences.
Supplementary Table 4: Inter-specific distances of the *bcd* intron 2.
Supplementary Figure 1: The bicoid genomic structures of Drosophila melanogaster, Drosophila simulans and Musca domestica.
Supplementary Figure 2: Multiple alignments of the bcd homeodomain amino acid sequences in dipteran species.

## Figures and Tables

**Table 1:** Intraspecific distances of the partial coding sequences and intron 2 of the *bcd* gene*.

Species (abbreviation)	Coding sequence	Intron 2	*N*
(1) *Triceratopyga calliphoroides *(Cl)	0.003024	0.000000	14*
(2) *Aldrichina grahami *(Gr)	0.000000	0.000000	7
(3) *Calliphora lata *(La)	0.001335	0.000000	10
(4) *Calliphora vicina *(Vi)	0.001661	0.000000	11
(5) *Chrysomya megacephala *(Mg)	0.000000	0.000000	3
(6) *Chrysomya pinguis *(Pi)	0.008341	0.055927	7*
(7) *Phormia regina *(Re)	0.000600	0.000000	8
(8) *Lucilia ampullacea *(Am)	0.008057	0.017557	3
(9) *Lucilia caesar* (Ca)	0.001029	0.000000	8
(10) *Lucilia illustris *(Il)	0.000000	0.000000	22
(11) *Hemipyrella ligurriens *(Li)	0.000000	0.000000	2
(12) *Lucilia sericata *(Se)	0.001468	0.013168	8*

Coding sequences = 3′ of exon 2 and 5′ half of exon 3 of *bcd.*

*N*: sample size.

*The number of the sequences deposited in GenBank is greater than the sample size.

**Table 2:** The *bcd* intron 2 sequences of 12 blowfly species: *Drosophila melanogaster* and *Musca domestica*.

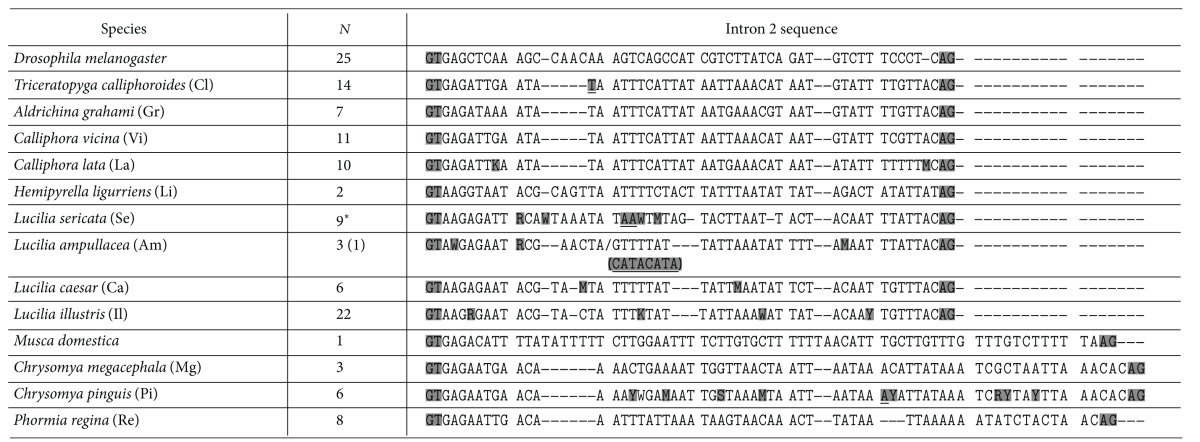

Single-nucleotide polymorphism: R = A or G; Y = C or T; W = A or T; M = A or C; K = G or T; M = A or C.

The underlined nucleotide(s), for example, AA stand for the two alleles of AA versus deletion (-).

*N*: sample size.

*For the intron 2 analysis, an additional sequence of the *bcd* gene in *L. sericata* (GU979856) was included.

*L. ampullacea* had an eight-nucleotide insertion in one individual at position 21 (indicated with slash symbol (/)).
