# Supplementary material for: Using the Developmental Gene Bicoid to Identify Species of Forensically Important Blowflies (Diptera: Calliphoridae)
Source: Biomed Res Int. 2013 Mar 18;2013:538051. doi: 10.1155/2013/538051 (PMC3613069; doi:10.1155/2013/538051)
Supplement: Supplementary file 1 — Supplementary Table 1: The primers used for the initial and second PCR amplification. Supplementary Table 2: The species-specific primer pairs used. Supplementary Table 3: Inter-specific distances of the bcd coding sequences. Supplementary Table 4: Inter-specific distances of the bcd intron 2. Supplementary Figure 1: The bicoid genomic structures of Drosophila melanogaster, Drosophila simulans and Musca domestica. Supplementary Figure 2: Multiple alignments of the bcd homeodomain amino acid sequences in dipteran species. [file 538051.f1.docx]

Supplementary Table 1. The primers used for the initial and second PCR amplification.

| Name | Sequence | Site* |
| --- | --- | --- |
| F1 | GGTGCAATCAA**Y**TA**Y**AA | 130~146 |
| R1 | TCCGATTG**D**AT**Y**TT**R**TG | 385~401 |
| F2 | **V**TCCGA**Y**TC**RY**TGGTTATGC | 195~214 |
| F3 | TC**RY**TGGTTATGCGTCG | 202~218 |
| R2 | GTCGGTGT**N**GT**N**GG**N**GT | 610~626 |

*Numbers correspond to the nucleotide positions in the *bcd* gene sequence of *Calliphora vicina* (AJ 297855.2)

IUPAC nucleotide ambiguity codes: Y= C or T; R= A or G; D= A or G or T; V = A or C or G; N= A or C or G or T

Supplementary Table 2. The species-specific primer pairs used.

| Species (abbreviation) | Primer pair |
| --- | --- |
| *Aldrichina grahami* (Gr) | FD1-RD1 |
| *Calliphora vicina* (Vi) | FD2-RD1 |
| *Calliphora lata* (La) | FD3-RD1 |
| *Triceratopyga calliphoroides* (Cl) | FD4-RD1 |
| *Lucilia ampullacea* (Am) | FD5-RD2 |
| *Lucilia caesar* (Ca) | FD6-RD1 |
| *Lucilia illustris* (Il) | FD6-RD1 |
| *Lucilia sericata* (Se) | FD6-RD1 |
| *Hemipyrella ligurriens* (Li) | FD7-RD1 |
| *Chrysomya megacephala* (Mg) | FD8-RD3 |
| *Chrysomya pinguis* (Pi) | FD8-RD3 |
| *Phormia regina* (Re) | FD9-RD1 |

* FD1 =AATTATATACGTCCATATATTCCAA, FD2 =AATTATATTCGCCCATATATTCCAA, FD3 =AATTATATACGCCCATATATTCCAA, FD4 =AATTATATACGCCCTTATATTCCAA, FD5 =AACTATATTCGACCATATATTCCAA, FD6 =AACTATATACGACCATATATTCCAA, FD7 =AACTATATACGCCCATATATTCCAA, FD8 =AATTATATACGTCCTTACATACCAA, FD9 =AATTATATACGTCCTTATATTCCAA, RD1 =GTTGGAGAGGGTGTTAATG, RD2 =GTTGGAGAGGGAGTTAATG, RD3 =GTTGGAGAGGGCGTTAATG

Supplementary Table 3. Inter-specific distances of the *bcd* coding sequences.*

| Species (abbreviation) | 1 | 2 | 3 | 4 | 5 | 6 | 7 | 8 | 9 | 10 | 11 |
| --- | --- | --- | --- | --- | --- | --- | --- | --- | --- | --- | --- |
| 1. *Triceratopyga calliphoroides* (Cl) |  |  |  |  |  |  |  |  |  |  |  |
| 2. *Aldrichina grahami* (Gr) | 0.028064 |  |  |  |  |  |  |  |  |  |  |
| 3. *Calliphora vicina* (Vi) | 0.024852 | 0.026070 |  |  |  |  |  |  |  |  |  |
| 4. *Calliphora lata* (La) | 0.019650 | 0.020765 |  |  |  |  |  |  |  |  |  |
| 5. *Hemipyrella ligurriens* (Li) | 0.084493 | 0.089250 | 0.090982 | 0.082406 |  |  |  |  |  |  |  |
| 6. *Lucilia sericata* (Se) | 0.098585 | 0.099813 | 0.104535 | 0.095777 | 0.040212 |  |  |  |  |  |  |
| 7. *Lucilia ampullacea* (Am) | 0.086292 | 0.086736 | 0.089432 | 0.084359 | 0.030217 | 0.044454 |  |  |  |  |  |
| 8. *Lucilia caesar* (Ca) | 0.090127 | 0.092777 | 0.091600 | 0.088529 | 0.025003 | 0.035779 | 0.024148 |  |  |  |  |
| 9. *Lucilia illustris* (Il) | 0.089464 | 0.092112 | 0.090937 | 0.087867 | 0.024387 | 0.035155 | 0.023532 | 0.000600 |  |  |  |
| 10. *Chrysomya megacephala* (Mg) | 0.107107 | 0.111463 | 0.121509 | 0.107017 | 0.073237 | 0.080998 | 0.076979 | 0.079292 | 0.078635 |  |  |
| 11. *Chrysomya pinguis* (Pi) | 0.108996 | 0.113382 | 0.123481 | 0.108907 | 0.076137 | 0.081311 | 0.079950 | 0.079598 | 0.078940 | 0.016460 |  |
| 12. *Phormia regina* (Re) | 0.090945 | 0.089625 | 0.099629 | 0.085363 | 0.068237 | 0.070626 | 0.074585 | 0.074244 | 0.073592 | 0.075238 | 0.073044 |

*coding sequences = 3’ of exon 2 and 5’ half of exon 3 of *bcd*

Supplementary Table 4. Inter-specific distances of the *bcd* intron 2.

| Species (abbreviation) | 1 | 2 | 3 | 4 | 5 | 6 | 7 | 8 | 9 | 10 | 11 |
| --- | --- | --- | --- | --- | --- | --- | --- | --- | --- | --- | --- |
| 1. *Triceratopyga calliphoroides* (Cl) |  |  |  |  |  |  |  |  |  |  |  |
| 2. *Aldrichina grahami* (Gr) | 0.054132 |  |  |  |  |  |  |  |  |  |  |
| 3. *Calliphora vicina* (Vi) | 0.025806 | 0.081759 |  |  |  |  |  |  |  |  |  |
| 4. *Calliphora lata* (La) | 0.083146 | 0.083146 | 0.111648 |  |  |  |  |  |  |  |  |
| 5. *Hemipyrella ligurriens* (Li) | 0.691219 | 0.691219 | 0.739435 | 0.703619 |  |  |  |  |  |  |  |
| 6. *Lucilia sericata* (Se) | 0.801229 | 0.801229 | 0.855949 | 0.818096 | 0.716021 |  |  |  |  |  |  |
| 7. *Lucilia ampullacea* (Am) | 0.652152 | 0.652152 | 0.700433 | 0.663752 | 0.332192 | 0.248790 |  |  |  |  |  |
| 8. *Lucilia caesar* (Ca) | 0.725626 | 0.725626 | 0.725626 | 0.654994 | 0.431641 | 0.282613 | 0.091903 |  |  |  |  |
| 9. *Lucilia illustris* (Il) | 0.649321 | 0.649321 | 0.649321 | 0.585513 | 0.380232 | 0.326756 | 0.093942 | 0.026332 |  |  |  |
| 10. *Chrysomya megacephala* (Mg) | 0.540786 | 0.681269 | 0.540786 | 0.549458 | 0.536573 | 0.729845 | 0.575563 | 0.662946 | 0.592222 |  |  |
| 11. *Chrysomya pinguis* (Pi) | 0.531879 | 0.671371 | 0.531879 | 0.535749 | 0.573629 | 0.719373 | 0.556632 | 0.624378 | 0.556850 | 0.031947 |  |
| 12. *Phormia regina* (Re) | 0.777166 | 0.970299 | 0.829592 | 0.792708 | 0.649321 | 0.850209 | 0.673514 | 0.867244 | 0.777166 | 0.271466 | 0.282675 |
