# Supplementary figures and images for: Using the Developmental Gene Bicoid to Identify Species of Forensically Important Blowflies (Diptera: Calliphoridae)
Source: Biomed Res Int. 2013 Mar 18;2013:538051. doi: 10.1155/2013/538051 (PMC3613069; doi:10.1155/2013/538051)

*Drosophila melanogaster*

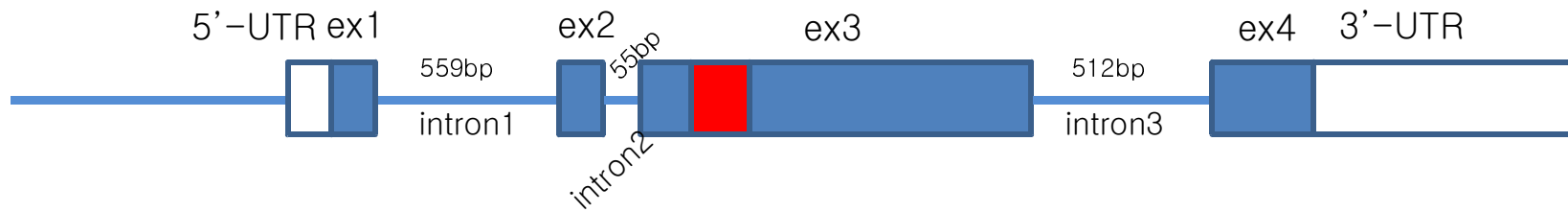

*Drosophila simulans*

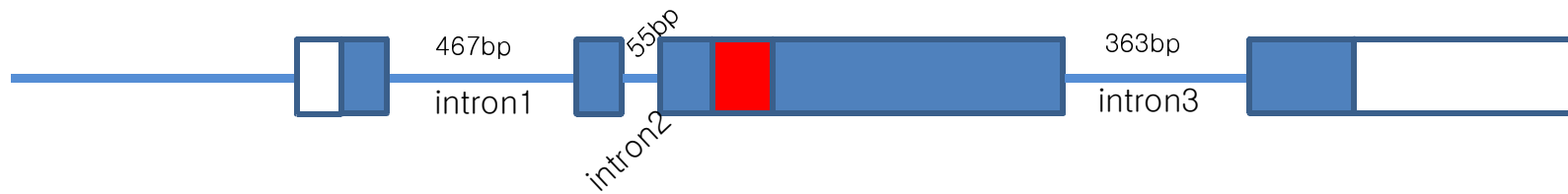

*Musca domestica*

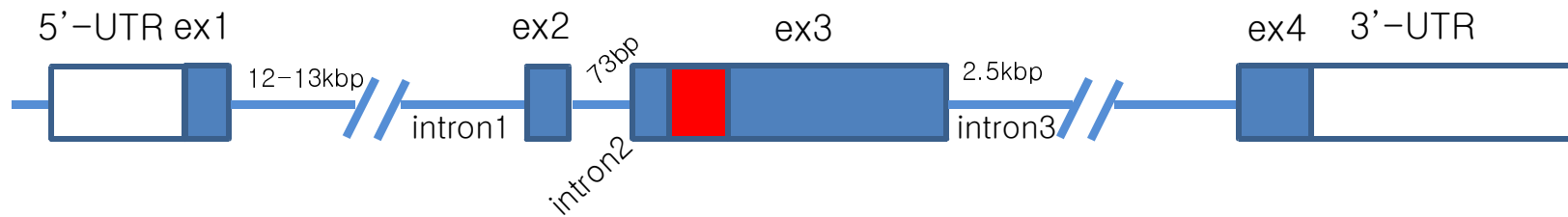

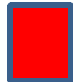 : homeobox

Supplement: Supplementary file 2 [file 538051.f2.pdf]
