# Supplementary material for: Using the Developmental Gene Bicoid to Identify Species of Forensically Important Blowflies (Diptera: Calliphoridae)
Source: Biomed Res Int. 2013 Mar 18;2013:538051. doi: 10.1155/2013/538051 (PMC3613069; doi:10.1155/2013/538051)
Supplement: Supplementary file 3 [file 538051.f3.pdf]

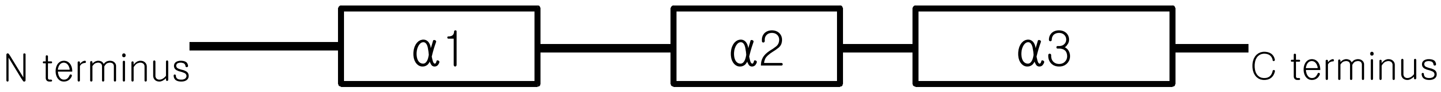

Megaselia abdita	RRRTRTTFTS	SQIAELEEYF	RQGKYLNNIR	LSELTGRLNL	GQAQVKIWFK	NRRRRFKIEQ
Drosophila melanogaster	RRRTRTTFTS	SQIAELEQHF	LQGRYLTAPR	LADLSAKLAL	GTAQVKIWFK	NRRRRHKIQS
Musca domestica	RRRTRTTFTS	AQIAELEQHF	LQGRYLTSSR	LAELSAKLTL	GTAQVKIWFK	NRRRRHKIQS
Aldrichina grahami	....	....	....	........A.	....	....
Lucilia illustris	....	....	....	........A.	....	....
Lucilia caesar	....	....	....	........A.	....	....
Lucilia ampullacea	....	....	....	.....V..A.	....	....
Lucilia sericata	....	....	....	........A.	....	....
Triceratopyga calliphoroides	....	....	....	........G.	....	....
Chrysomya megacephala	....	....	....	........A.	....	....
Chrysomya pinguis	....	....	....	........A.	....	....
Calliphora vicina	....	....	....	........A.	....	.........A
Phormia regina	....	....	....	........A.	....	....
Calliphora lata	....	....	....	........A.	....	....
Hemipyrellia ligurriens	....	....	....	........A.	....	....

Suppl Fig. 2
